# Dietary and lifestyle quality and endometrial cancer: Incidence and survivorship outcomes—An umbrella review of systematic reviews and meta-analyses

**DOI:** 10.1371/journal.pone.0360238

**Published:** 2026-10-14

**Authors:** Ruqaiya Al Ramadhani, Newshaw Arvin, Saphy Moussa, Monika Janda, Mary C. Playdon, Andreas Obermair

**Affiliations:** 1 Centre for Health Services Research, Faculty of Health, Medicine and Behavioural Sciences, The University of Queensland, Brisbane, QLD, Australia; 2 School of Medicine, The University of Queensland, Brisbane, QLD, Australia; 3 School of Public Health, The University of Queensland, Brisbane, QLD, Australia; 4 Department of Nutrition and Integrative Physiology, The University of Utah, Salt Lake City, Utah, United States of America; 5 Cancer Control and Population Sciences, Huntsman Cancer Institute, University of Utah, Salt Lake City, Utah, United States of America; 6 Queensland Centre for Gynaecological Cancer Research, The University of Queensland, Brisbane, QLD, Australia; Teikyo University: Teikyo Daigaku, JAPAN

## Abstract

Composite dietary and lifestyle indices capture the combined effects of multiple health-related behaviours and may better reflect real-world cancer risk and survivorship than single exposures. This umbrella review aimed to evaluate and grade the strength of evidence for associations between adherence to composite dietary and lifestyle quality indices and endometrial cancer (EC) outcomes, including risk, EC-specific survival, overall survival, disease recurrence, quality of life, and new-onset cardiometabolic diseases. PubMed, Embase, Scopus, Web of Science, CINAHL, and Cochrane Library databases were systematically searched for relevant systematic reviews. Evidence was graded using prespecified umbrella review criteria. A total of 21 systematic reviews were included. Nine investigated diet quality (7 meta-analyses) and 12 investigated lifestyle exposure or interventions (8 meta-analyses). The outcomes assessed were EC risk (N = 14), all-cause or cancer-specific mortality (N = 5), recurrence (N = 4), overall or cancer-specific survival (N = 3), QoL (N = 5), and cardiometabolic events (N = 1). Following a Mediterranean diet was associated with 29% lower odds of EC (pooled OR = 0.71, 95% CI 0.51–0.98; class IV), whereas Western dietary patterns were associated with 20% higher odds of EC (pooled OR = 1.20, 95% CI 1.03–1.39; class IV). A combined healthy diet and lifestyle approach was associated with a 46% lower hazard of EC incidence (pooled HR = 0.54, 95% CI 0.42–0.71; class III), although heterogeneity was high. No significant associations were observed between lifestyle interventions and global or domain-specific quality of life (QoL) across follow-up durations ranging from 9 weeks to 12 months, irrespective of the validated assessment instruments used. Evidence remains limited regarding the associations between diet and lifestyle factors and EC recurrence, survival, mortality, and cardiometabolic outcomes. Our umbrella review identified suggestive (class III–IV) evidence for associations between healthy dietary patterns, particularly the Mediterranean diet, and combined healthy lifestyle approaches with lower EC incidence. Evidence remains insufficient or non‐significant for post-diagnosis outcomes, underscoring the need for further high-quality research.

## Introduction

Endometrial cancer (EC) is the sixth most common cancer among females worldwide [1]. Women diagnosed with early-stage EC generally have a good prognosis, with 5-year survival exceeding 90% [2]. However, their risk of cardiometabolic diseases, cardiovascular death, and other genitourinary conditions is elevated compared with cancer-free females, even when matched for the level of obesity [3–6]. Age-adjusted incidence and mortality rates related to EC are highest in countries with higher human development index (HDI) levels [1]. The epidemic of obesity in those regions is thought to be a major reason for the observed increase in EC rates [1]. Yet, increased incidence of and mortality from non-endometrioid ECs that are less obesity-driven but more lethal across racial/ethnic groups suggests other aetiology may be involved as well [7,8].

Currently, no standard screening test is available for the early detection of EC in asymptomatic women, making cancer prevention strategies essential. While evidence suggests that adherence to healthy dietary patterns and lifestyle behaviours may reduce EC risk, findings have been mixed [9]. The World Cancer Research Fund (WCRF) Continuous Update Project (CUP) identifies energy imbalance, particularly excess body adiposity, as a convincing EC risk factor [10], with chronic inflammation, elevated estrogen, and hyperinsulinemia proposed as underlying mechanisms [11–14]. Independent of obesity, high dietary glycemic load is associated with increased EC risk, while physical activity and coffee intake appear protective [10]. A recent umbrella review of 171 meta-analyses reinforced these findings, highlighting strong evidence for obesity and suggestive evidence for physical activity and coffee intake [15]. However, that review focused on individual risk factors such as specific foods, nutrients, or behaviours rather than evaluating the cumulative effect of overall diet quality or lifestyle patterns. As a result, evidence for broader dietary patterns and lifestyle combinations remains limited and inconclusive [10,15]. In contrast, our umbrella review investigates the collective influence of diet quality indices and lifestyle scores, which reflect adherence to comprehensive health guidelines and capture the synergistic effects of multiple behaviours. This approach provides a more integrated understanding of how combined lifestyle factors relate to EC risk and other outcomes. While certain foods with inflammatory or oxidant properties (e.g., fast food, red and processed meats) have been linked to EC development [16,17], and physical activity contributes to cancer prevention through metabolic regulation [18,19], specific post-diagnosis guidelines for EC survivors remain lacking. Current recommendations largely mirror those for EC prevention [10].

Although several systematic reviews have assessed individual dietary or lifestyle components in relation to EC risk [20–31], fewer have examined composite indices or cancer prevention guidelines [32–38]. Moreover, most focused solely on EC incidence, with limited attention to outcomes following diagnosis, such as recurrence, survival, quality of life (QoL), or cardiometabolic conditions. This umbrella review therefore seeks to synthesise current evidence on the association between adherence to diet quality or lifestyle guidelines and key EC outcomes. It also aims to critically appraise the strength and consistency of the existing evidence base, identify methodological limitations, and highlight priority areas for future research. The primary objective of this umbrella review was to evaluate the association between adherence to composite indices of dietary and lifestyle quality, and the incidence of EC. Secondary objectives were to assess associations with post-diagnosis outcomes, including recurrence, overall and cancer-specific survival, QoL, and cardiometabolic outcomes. Prespecified indices included commonly used dietary and lifestyle indices, such as the Mediterranean Diet Score (MDS), Healthy Eating Index (HEI), Alternative Healthy Eating Index (AHEI), Dietary Approaches to Stop Hypertension (DASH), World Cancer Research Fund/American Institute for Cancer Research (WCRF/AICR) score, American Cancer Society (ACS) guidelines, and Healthy Lifestyle Index. We hypothesised that higher adherence to healthier dietary and lifestyle indices would be associated with lower EC incidence, while associations with post-diagnosis outcomes were anticipated to be uncertain given limited and heterogeneous evidence.

## Methods

This systematic umbrella review followed the Preferred Reporting Items for Systematic Reviews and Meta-Analyses (PRISMA) statement [39] (see Supplementary Checklist S5 in S1 File. PRISMA 2020 checklist). The protocol was registered in PROSPERO (CRD42022312161) on 1 April 2022. No major deviations from the registered protocol occurred, except that no language restrictions were ultimately applied.

### Search strategy

A systematic search of electronic databases, including PubMed, Embase, Cochrane Library, Scopus, CINAHL, and Web of Science from inception to the 3^rd^ of December 2024 was conducted. The key search terms for exposure included ‘diet*’ OR ‘diet score’ OR ‘diet index’ OR ‘diet quality’ OR ‘dietary guidelines’ OR ‘dietary pattern’ OR ‘dietary change’ OR ‘dietary intervention’ OR ‘dietary modification’ OR ‘lifestyle*’ OR ‘lifestyle modification’ OR ‘lifestyle intervention’ OR ‘healthy lifestyle’. Additionally, phrases like ‘cancer prevention guidelines,’ ‘American Cancer Society,’ and ‘World Cancer Research Fund,’ were incorporated. Outcome terms included ‘endometrial’ or ‘uterine cancer,’ ‘carcinoma,’ ‘tumo(u)r,’ ‘neoplasm,’ and ‘malignancy.’ Filters for ‘systematic review’ and ‘meta-analysis’ were also applied. Additionally, the bibliography of included articles and relevant protocols registered at PROSPERO were searched. Detailed search terms used across different databases are presented in Supplementary Table S1 in S1 File.

### Selection criteria

Inclusion criteria: A pre-defined list of criteria was used to include systematic reviews with or without meta-analyses evaluating the association between adherence to dietary and/or lifestyle recommendations and EC outcomes.

The PICOS components of this review’s research question are:

#### Population.

Women aged 18 years or older, cancer-free or diagnosed with EC.

#### Intervention/exposure.

1) Diet quality indices including Healthy Eating Index (HEI), Alternative Healthy Eating Index (AHEI), Australian Dietary Guidelines, Mediterranean diet score (MDS), alternate Mediterranean diet score (aMDS), Dietary Approaches to Stop Hypertension (DASH), data-driven dietary patterns (e.g., Western/Prudent), or healthy eating dietary intervention that manipulates more than one food exposure.
2) Lifestyle indices including the American Cancer Society (ACS) Guidelines on nutrition and physical activity for cancer prevention, World Cancer Research Fund/ American Institute for Cancer Research (WCRF/AICR) score, and Healthy Lifestyle index. These indices incorporate key lifestyle factors, including body mass index (BMI), physical activity, smoking status, and dietary intake. Lifestyle interventions typically involve a combination of healthy dietary modifications and increased physical activity.

For interventional studies, there were no limits placed on the setting (electronic, telephone or in-person delivery) or the duration and frequency of the intervention.

#### Comparison.

High versus low score (observational); No therapeutic/preventive measures or attention control (interventional).

#### Outcome.

The primary outcome is EC risk. Secondary outcomes include cancer-specific survival, overall survival, disease recurrence or progression, quality of life, and new-onset cardiometabolic diseases such as hypertension, acute cardiovascular events (stroke, MI), dyslipidaemia, or type 2 diabetes.

#### Study design.

All systematic reviews with or without meta-analyses of RCTs, cohort, or case-control studies.

Exclusion criteria: Reviews were excluded if they were not systematic reviews or did not focus on human studies. Additionally, if two systematic reviews addressed the same research question using the same primary studies, the most recent or most comprehensive review was included. Although our initial protocol noted the exclusion of non-English reviews, no language filters were applied during the database searches. All included reviews were published in English, reflecting the available literature rather than an enforced exclusion. Reasons for full-text exclusions are reported in the Results.

### Data extraction

Screening of the articles for inclusion was conducted in two steps by two independent reviewers (RA, and NA or SM) at each stage: 1) Screening of titles and abstracts; 2) Full-text review of studies that met the inclusion criteria. Discrepancies between the reviewers were resolved by consensus. Two independent reviewers (RA, and NA or SM) extracted the following data: Author, year, research question, databases and timeframe searched, inclusion/exclusion criteria, primary articles of each review (study numbers, study design, author, year, sample size, age of participants, diet/lifestyle measurement method, mean follow-up), main outcomes including effect estimates of primary studies or pooled effects (for meta-analyses) with respective 95% confidence intervals (CI) and p-values. Data were summarised in an evidence table incorporating all the details listed above.

### Quality assessment

The methodological quality of included studies was assessed by two independent reviewers (RA, and NA or SM) using AMSTAR 2, which is validated and reliable [40,41]. AMSTAR 2 is a critical appraisal tool for systematic reviews that include randomised or non-randomised studies of healthcare interventions, or both. This is a modified version of AMSTAR and comprises 16 items with an overall quality rating ranging from high or moderate to low, or critically low. AMSTAR 2 results for each included article are presented in a summary table. Disagreements in quality rating were resolved by consensus.

### Data synthesis

For each systematic review, a descriptive analysis was conducted. A structured summary table was formatted with reviews being grouped according to the exposure/ intervention into 1) diet quality indices/patterns, and 2) lifestyle indices/patterns. For each meta-analysis, summary effect sizes with the corresponding 95% CI and p-values were recomputed by fitting a series of random-effects models using the ‘metaumbrella’ package in R (version 4.0.4) (2021) [42]. All metaumbrella functions in this package require well-formatted datasets containing mandatory variables for each primary study that had been included in previous meta-analyses. Those mandatory variables include “meta-review”, an identifier for the source of the meta-analysis, “factor”, a factor variable which is the intervention/exposure studied, and “author”, “year”, and “measures of effect sizes” for each primary study. The measures of effect sizes in this umbrella review were hazard ratios (HR), odds ratios (OR), and mean differences. Additional required information for each individual study, depending on the effect size measures, was the value of the effect size, the lower and upper bounds of the 95% CI, the total number of participants, the number of cases, the number of controls, the number of exposed participants (exposed cases/controls), and the number of non-exposed participants (non-exposed cases/controls). Continuous outcomes were synthesised as standardised mean differences (SMDs) expressed as Hedges g. Quality of life (QoL) outcomes were assessed using validated instruments reported in the included trials, including the Functional Assessment of Cancer Therapy–General (FACT-G), the Functional Assessment of Cancer Therapy–Fatigue (FACT-F), and the 12-Item Short Form Health Survey (SF-12). Where multiple QoL domains (e.g., physical, emotional, social, fatigue) or time points (e.g., 3 and 6 months) were reported, these were treated as separate outcomes in the synthesis. The Hedges g effect measure was interpreted based on benchmarks developed by Cohen (1988) [43], where values of 0.2, 0.5, and 0.8 were considered small, medium, and large effects, respectively. For the exposure Mediterranean diet score (MDS) and EC risk outcome, primary studies reported different effect measures. Case-control studies reported odds ratios, whereas a prospective cohort study by George et al. [44] reported hazard ratios. Because these measures are not directly comparable, we did not pool them. Accordingly, design‑ and effect‑measure–stratified sensitivity analyses were undertaken: case-control studies reporting odds ratios were synthesised quantitatively, while cohort evidence was examined separately and narratively. The cohort study by George et al. [44] was therefore excluded from the pooled meta‑analysis but retained for sensitivity assessment. The case-control study by Dalvi et al [45] was excluded due to insufficient data for quantitative synthesis. Additionally, two studies (1 cohort and 1 case-control) were excluded from analyses of Prudent and Western dietary patterns and EC incidence due to insufficient data [46,47]. Moreover, we pooled mean differences from two RCTs measuring QoL using SF-12 physical health on lifestyle intervention at 6-months [48,49]. Those RCTs were included narratively in an updated systematic review with no quantitative synthesis [50]. For one of the pooled RCTs by Haggerty et al. [48], we converted medians and quartile ranges to means and SDs based on the method by McGrath (2020) [51], as this study reported median changes and quartiles only. From the latter RCT, two QoL estimates from two different lifestyle intervention groups that shared a common control group were pooled while adjusting for the shared control group. The umbrella function in the ‘metaumbrella’ package automatically extracted data, performed meta-analyses and additional tests needed: tests for estimating heterogeneity, 95% prediction interval (PI), excess significance bias (ESB), and small-study effects. The Restricted Maximum Likelihood (REML) method was used to estimate the I^2^ statistics of heterogeneity. I^2^ values of 25%, 50%, and 75% indicated low, moderate, and high heterogeneity, respectively. Egger’s regression asymmetry test was utilised for estimating small-study effects with a p-value < 0.05 indicating statistical significance. In line with methodological recommendations, results for small-study effects (Egger’s test) and excess significance bias were interpreted and reported only for meta-analyses including ≥10 primary studies. ESB assesses whether the observed number of statistically significant studies (p < 0.05) exceeds the expected number, with a p-value of < 0.05 indicating evidence of ESB. Forest plots were presented for visualisation of summary effect sizes. All statistical analyses were conducted using ‘metaumbrella’ package in R (version 4.0.4) (2021).

### Certainty of evidence

The evidence generated was stratified according to a pre-specified criterion “Ioannidis” described by Fusar-Poli and Radua (2018) that rates evidence as convincing (class I) when the number of cases is > 1000, p < 10^−6^, I^2^ < 50%, the 95% prediction interval excludes the null, there are no small‐study effects, and no ESB; highly suggestive (class II) when number of cases is > 1000, p < 10^−6^, the largest study has a statistically significant effect, and class I criteria are not met; suggestive (class III) when the number of cases is > 1000, p < 10^−3^, and class I‐II criteria are not met; weak (class IV) when p < 0.05 and class I‐III criteria are not met; and non‐significant when p ≥ 0.05 [52].

### Overlap assessment

A citation matrix was used to display the level of overlap in the primary articles [53]. The columns in the matrix are the included systematic reviews and the rows are the primary studies for each systematic review. If the outcomes assessed were different across systematic reviews with similar primary articles, then this was not considered overlapping. To quantify overlap, Corrected Covered Area (CCA) was calculated based on the formula: CCA= (N-r)/(rc-r), where N is the number of total primary studies included in systematic reviews, including double counting, r is the number of primary studies (number of rows), and C is the number of included systematic reviews (number of columns). The interpretation of CCA is: 0–5% slight overlap, 6–10% moderate overlap, 11–15% high overlap and >15% very high overlap [53]. To further customize overlap, CCAs for each pair of systematic reviews were also calculated and graphically represented with colour codes; slight (white), moderate (green), high (yellow), and very high (red) [54].

## Results

### Study selection

Our search identified 1652 records (Fig 1). After removing duplicates and ineligible reviews based on screening titles and abstracts, 56 reviews were retrieved in full text. Of these, 35 were excluded for the following reasons: 5 were duplicates [55–59], 10 did not meet study design criteria [60–69], 1 wrong population [70], 7 did not meet the criteria for intervention or exposure [71–77], 7 did not include our outcomes of interest [56,58,78–82], 1 abstract [83], 2 protocols [84,85], 1 old version of an updated review [57], and 1 was an update of an included systematic review with the same primary articles and results for EC outcomes [86]. The update was excluded because the previous meta-analysis was more comprehensive and the update included additional cancer sites without adding new evidence for EC outcomes. A total of 21 eligible systematic reviews were included in the data synthesis. The exposure or intervention was diet quality indices/patterns in 9 systematic reviews [33–35,87–92] (7 meta-analyses) and lifestyle indices/patterns in 12 systematic reviews [32,36–38,50,76,93–98] (8 meta-analyses). The characteristics of the included systematic reviews and meta-analyses were summarised in Supplementary Table S2 in S1 File.

**Fig 1:**
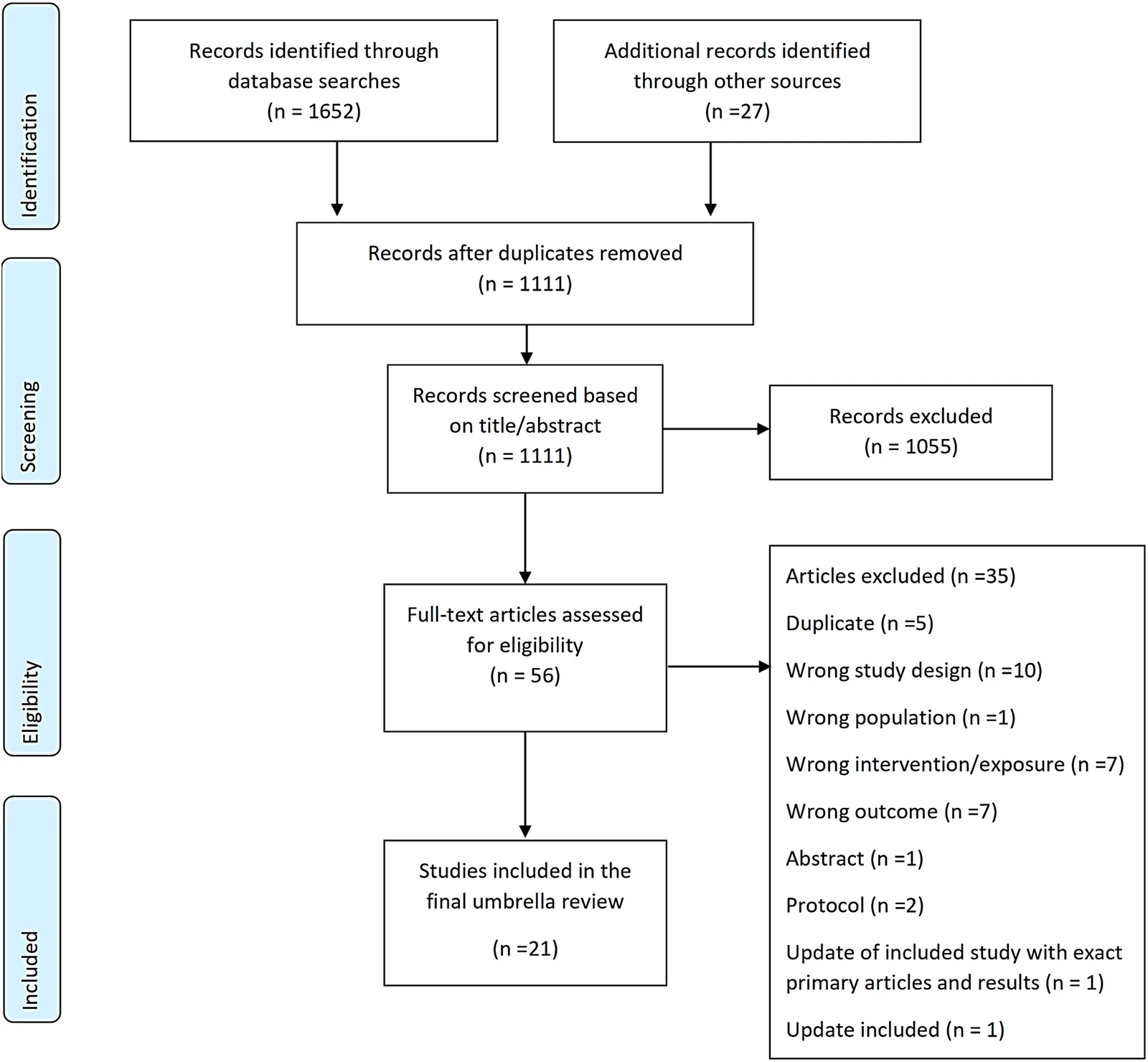
PRISMA flow diagram of study selection. The diagram presents the number of records identified, screened, excluded, and included in the umbrella review, with reasons for exclusion at the full-text stage. EC = endometrial cancer.

### Quality assessment

The quality of the included systematic reviews was moderate [33,36,91] to high [32,34,35,38,50,97,98] in ten studies and low [76,87,89] to critically low [37,90,93–96] in 11 studies. Supplementary Table S3 in S1 File shows the AMSTAR 2 quality assessment domains and ratings for each systematic review. The main reasons for scoring low or critically low were failure to explicitly report that methods were established prior to conducting the review (6/11 systematic reviews), no explanation for the selection of study designs for inclusion (7/11 systematic reviews), and not listing the excluded studies and/or justification for the exclusion (8/11 systematic reviews). Overall, the lowest score obtained was for the funding source of primary studies, which was not reported in 18/21 systematic reviews.

### Quantitative synthesis

For the quantitative synthesis, we re-analyzed 4 of 14 meta-analyses (Table 1). The remaining 10 meta-analyses had overlapping primary studies with the 4 re-analysed meta-analyses. We therefore included the updated or most comprehensive meta-analyses, which mostly had higher quality ratings, in our quantitative synthesis. Key findings for each EC outcome of interest are presented in Table 2, listing the number of high-quality individual studies for each outcome.

**Table 1:** Quantitative summary of meta-analyses on diet quality and lifestyle and EC outcomes.

Exposure/ Intervention	Outcome	Effect measure	No. of studies	Sample size	Cases	Random Effect (95% CI)	*P*	*I*^*2* (^%)	95% PI ^ᵻ^	Largest study significant^a^	Level of evidence
Mediterranean diet	EC risk	OR	2	5,383	1708	0.71 (0.51, 0.98)	0.036	44.7%	PI not estimable	Yes (Filomeno et al., 2015)	IV
Dietary patterns											
Prudent	EC risk	OR	3	3,798	1,448	0.89 (0.76, 1.05)	0.170	0%	PI not estimable	Yes (Biel et al., 2011)	NS
Western	EC risk	OR	3	3,798	1,448	1.20 (1.03, 1.39)	0.022	0%	PI not estimable	No (Biel et al., 2011)	IV
Combined lifestyle	EC risk	HR	5	597,047	4,548	0.54 (0.42, 0.71)	<0.001	86%	PI not estimable	Yes (Romaguera et al., 2012)	III
Lifestyle intervention	QoL-Global at 3 months (FACT-G)	Hedge’s g	2	120	64	0.16 (−0.20, 0.52)	0.372	0%	PI not estimable	No (McCarroll et al, 2014)	NS
	QoL-Global at 6 months (FACT-G)	Hedge’s g	2	120	64	0.18 (−0.18, 0.54)	0.332	0%	PI not estimable	No (McCarroll et al, 2014)	NS
	QoL-Physical at 3 months (FACT-G)	Hedge’s g	2	120	64	0.07 (−0.29, 0.43)	0.688	0%	PI not estimable	No (McCarroll et al, 2014)	NS
	QoL-Physical at 6 months (FACT-G)	Hedge’s g	2	120	64	0.20 (−0.19, 0.58)	0.320	0%	PI not estimable	No (McCarroll et al, 2014)	NS
	QoL-Emotion at 3 months (FACT-G)	Hedge’s g	2	120	64	0.07 (−0.29, 0.43)	0.704	0%	PI not estimable	No (McCarroll et al, 2014)	NS
	QoL-Emotion at 6 months (FACT-G)	Hedge’s g	2	120	64	0.06 (−0.30, 0.42)	0.726	0%	PI not estimable	No (McCarroll et al, 2014)	NS
	QoL-Social at 3 months (FACT-G)	Hedge’s g	2	120	64	0.19 (−0.17, 0.55)	0.298	0%	PI not estimable	No (McCarroll et al, 2014)	NS
	QoL-Social at 6 months (FACT-G)	Hedge’s g	2	120	64	0.22 (−0.14, 0.58)	0.228	0%	PI not estimable	No (McCarroll et al, 2014)	NS
	QoL-Fatigue at 3 months (FACT-F)	Hedge’s g	2	120	64	0.11 (−0.25, 0.47)	0.559	0%	PI not estimable	No (McCarroll et al, 2014)	NS
	QoL-Fatigue at 6 months (FACT-F)	Hedge’s g	2	120	64	0.08 (−0.28, 0.44)	0.653	0%	PI not estimable	No (McCarroll et al, 2014)	NS
	SF-12 Physical at 6 months	Hedge’s g	2*	96	46	0.24 (−1.01, 1.50)	0.706	88%	PI not estimable	No (Zamorano et al, 2021)	NS

Abbreviations; N = number, I^2^ = Heterogeneity, PI = Prediction interval, EC = endometrial cancer, OR=odds ratio, HR = hazard ratio, NS = not significant, QoL = Quality of Life, FACT-G = Functional Assessment of Cancer Therapy – General, FACT-F = Functional Assessment of Cancer Therapy – Fatigue, SF-12 = 12‐item Short Form (SF‐12) Physical Health questionnaire.

ᵻ95% prediction intervals were not estimable due to the small number of studies included in each meta-analysis (2–5 studies).

^a^Largest study refers to the primary study with the largest total sample size (cases and controls), used as a proxy for statistical weight. Statistical significance is based on whether the 95% confidence interval excludes the null value.

*One study with two effect sizes for independent intervention groups was pooled, with adjustment for the shared control group.

Note: Tests for small-study effects (Egger’s test) and excess significance bias were not assessed, as all meta-analyses included fewer than 10 studies.

**Table 2:** Summary of associations between diet quality and lifestyle, and EC outcomes.

EC outcomes	Total N of primary studies re-analysed	High quality studies assessing diet quality*	High quality studies assessing lifestyle*	Results indicate diet quality important, Pooled estimate (95% CI)	Results indicate lifestyle important, Pooled estimate (95% CI)
Risk	10	4 out of 5	4 out of 5	(N = 2 studies on MDS) OR= 0.71 (0.51, 0.98) (N = 3 studies on Western dietary patterns) OR= 1.20 (1.03, 1.39)	(N = 5 studies) HR = 0.54, (0.42, 0.71)
Recurrence	–	–	–	–	–
Overall survival	1	–	–	–	NS
EC-specific survival	–	–	–	–	–
QoL-Global at 3 months (FACT-G)	2	–	–	–	NS
QoL-Global at 6 months (FACT-G)	2	–	–	–	NS
QoL-Physical at 3 months (FACT-G)	2	–	–	–	NS
QoL-Physical at 6 months (FACT-G)	2	–	–	–	NS
QoL-Emotion at 3 months (FACT-G)	2	–	–	–	NS
QoL-Emotion at 6 months (FACT-G)	2	–	–	–	NS
QoL-Social at 3 months (FACT-G)	2	–	–	–	NS
QoL-Social at 6 months (FACT-G)	2	–	–	–	NS
QoL-Fatigue at 3 months (FACT-F)	2	–	–	–	NS
QoL-Fatigue at 6 months (FACT-F)	2	–	–	–	NS
SF-12 Physical at 6 months	2	–	–	–	NS
Cardiometabolic events at 6 months	5^±^	–	–	–	NS
Cardiometabolic events at 12 months	2	–	–	–	NS

Abbreviations; N = number, CI = confidence interval, PA = physical activity, OR= odds ratio, HR = hazard ratio, NS = not statistically significant, QoL = Quality of Life, FACT-G = Functional Assessment of Cancer Therapy – General, FACT-F = Functional Assessment of Cancer Therapy – Fatigue, SF-12 = 12‐item Short Form (SF‐12) Physical Health questionnaire.

*Quality of primary studies retrieved from the included meta-analyses.

±Only one episode of congestive heart failure in one RCT. No cardiovascular events were recorded in the other 4 studies.

Systematic reviews and meta-analyses assessed five key outcomes: EC risk (14 reviews, 11 with meta-analyses), all-cause or cancer-specific mortality (7 reviews, 5 with meta-analyses), and disease recurrence (4 meta-analyses), Quality of life (QoL), including global and domain-specific measures (5 reviews, 3 with meta-analyses), and cardiometabolic events (1 meta-analysis).

### Findings and certainty of evidence

Better diet quality (measured by MDS) was significantly associated with lower odds of EC incidence [pooled OR=0.71, 95% CI (0.51–0.98), p = 0.036, I² = 44.7%]. In pooled case-control analysis, higher MDS was associated with lower odds of EC incidence. In contrast, the single available prospective cohort study reporting hazard ratios did not demonstrate a statistically significant association. Because only one cohort study was available, a pooled cohort analysis was not feasible; however, this design‑stratified comparison suggests that the observed inverse association is primarily driven by case-control evidence.

Prudent (healthy) dietary patterns suggested lower odds but lacked statistical significance, while Western patterns were significantly associated with higher EC odds [pooled OR=1.20, 95% CI (1.03–1.39), p = 0.022, I² = 0%]. Healthy lifestyles were associated with a lower risk of EC [pooled HR = 0.54, 95% CI (0.42–0.71), p=<0.001], however, substantial heterogeneity was observed (I² = 86%), suggesting variability across studies. Small-study effects and excess significance bias were not assessed, as all meta-analyses included fewer than 10 studies. 95% prediction intervals were not estimable for any meta-analysis due to the small number of studies (2–5 studies), indicating uncertainty in the observed effects. Evidence strength was suggestive (class III) for combined lifestyle, weak (class IV) for MDS and Western patterns, and non-significant for Prudent patterns. See Table 1 and Fig 2 for details.

**Fig 2:**
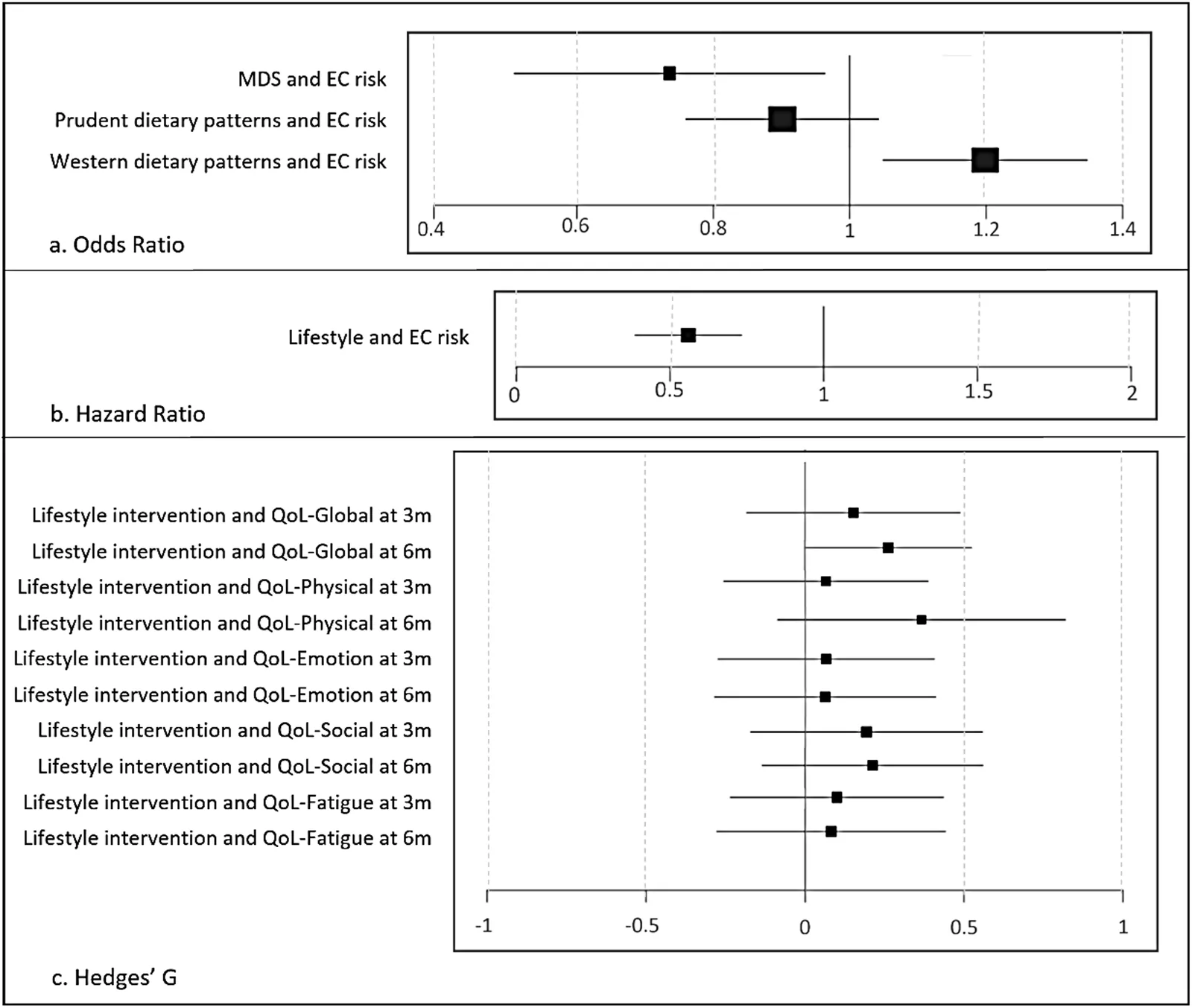
Forest plots of associations between diet quality and lifestyle and endometrial cancer (EC) outcomes. (a) Pooled odds ratios (OR) for dietary exposures and EC incidence; (b) pooled hazard ratios (HR) for lifestyle indices and EC incidence; and (c) standardised mean differences (Hedges’ g) for quality of life (QoL) outcomes. Horizontal lines represent 95% confidence intervals (CI). CI = confidence interval; OR = odds ratio; HR = hazard ratio.

We found no association between lifestyle interventions and global or domain-specific QoL (FACT-G), including physical, emotional, social wellbeing, and fatigue at 3- and 6-month follow-ups (meta-analysis could not be conducted for 9-week (FACT-G) and 12-month (FACT-G or SF-12 Physical/Mental Health questionnaire or 9-item Patient Health questionnaire) owing to limited data). In our analyses, we obtained small (around 0.2) and positive values of Hedges’ g for all QoL domains favouring the usual care group rather than the intervention group. However, all effects were statistically non-significant (Table 1). No evidence of heterogeneity was found, except for the association between lifestyle interventions and the SF-12 Physical score at 6 months (I² = 88%). The strength of evidence for QoL outcomes showed non-significant levels (Table 1).

No significant associations were identified between lifestyle interventions and other outcomes, including EC recurrence, mortality, and cardiometabolic events, due to a lack of primary studies reporting these outcomes in women with EC and/or no events occurring in studies evaluating these outcomes (Supplementary Table S2 in S1 File).

### Overlap of primary studies

The citation matrix revealed a moderate overall overlap of primary studies across systematic reviews (Corrected Covered Area (CCA) ~7%, Supplementary Table S4 in S1 File). For reviews on similar interventions and outcomes, 54 of 210 pairs showed a very high overlap (CCA ≥ 15%), with 6 pairs having 100% overlap (Fig 3).

**Fig 3:**
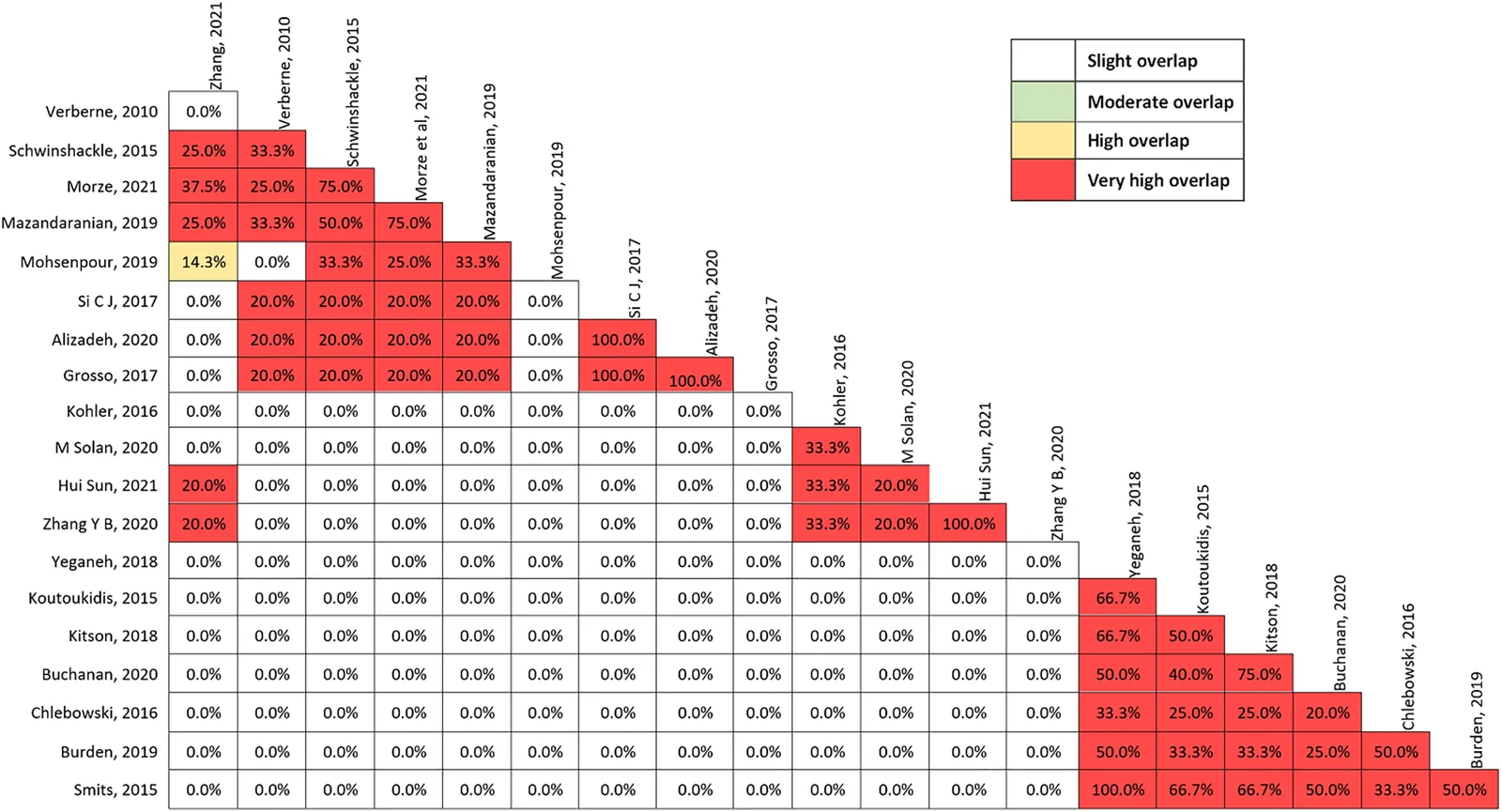
Corrected covered area (CCA) percentages for each pair of included systematic reviews. Higher values indicate greater overlap in primary studies between reviews. CCA = corrected covered area.

## Discussion

### Main findings

Most systematic reviews have predominantly addressed the risk of EC development, with limited focus on outcomes following diagnosis, such as disease recurrence, mortality, quality of life (QoL), and new cardiometabolic events [32–38]. This umbrella review examined the independent associations of diet quality and the combined influence of diet and lifestyle, considering their potential combined associations with EC outcomes. It also assessed their relationships with key clinical endpoints and the strength of the existing evidence. The findings suggest that better diet quality, as measured by MDS, and healthier lifestyle patterns are associated with lower odds of EC incidence, whereas Western dietary patterns are associated with higher odds. However, given that the strength of evidence is weak to suggestive (classes III–IV), these findings should be interpreted with caution.

Women in the highest category of MDS had 29% lower odds of EC than women in the lowest category. The Mediterranean diet has been linked to a lower risk of multiple chronic diseases, including cancer, and longevity based on studies conducted globally [99–101]. It is characterised by the intake of fruits, vegetables, nuts, whole grains, olive oil, moderate wine consumption, and limited intake of animal fat and red meat [102]. This dietary pattern consists of high amounts of fibre, antioxidants (particularly polyphenols), vitamins (E and C), and a balanced ratio of essential amino acids (omega-3 and omega-6) [102]. The age-standardised EC incidence rates in Mediterranean regions are around 3-fold lower compared to Northern America and Europe [103]. This may be related to lifestyle behaviours, including the traditional Mediterranean diet and a lower prevalence of obesity in those regions. The pooled effect estimate of MDS and EC incidence was based on two case-control studies and provided weak evidence of an inverse association. Unlike Morze et al.‘s meta-analysis [35], we did not pool results across differing study designs or effect measures. Consequently, the large prospective cohort study by George et al. [44], which reported hazard ratios, was not included in the meta-analysis. When examined separately in a design-stratified sensitivity analysis, this study did not show a statistically significant association. An additional case-control study by Dalvi et al. [45] was excluded due to insufficient data for quantitative synthesis. The two studies included in the pooled analysis, by Filomeno et al. [59] and Ricceri et al. [104], both reported statistically significant inverse associations between MDS and EC incidence, with the larger study by Filomeno contributing greater weight to the summary estimate. Taken together, these findings indicate that the observed inverse association is primarily supported by case-control evidence and should therefore be interpreted cautiously.

Our review showed that following a Western diet was associated with a 20% higher odds of EC. As opposed to a Mediterranean diet, Western-type diets comprise higher intakes of animal fat, red meat, sugar, and refined grains coupled with low intakes of fruits, vegetables, and whole grains [105]. Our analysis of three out of five studies on dietary patterns and EC risk (one cohort and two case-control studies) with complete data for analysis was consistent with the original meta-analysis by Alizadeh et al. [91]. Alizadeh and colleagues reported statistically significantly higher EC risk for the highest vs lowest categories of Western dietary patterns and, conversely, lower EC risk for individuals with healthier versus less healthy dietary patterns. Our results were consistent with these findings, but did not reach statistical significance for the latter association.

A growing body of evidence suggests that a healthy diet, optimal body weight for height, and higher levels of physical activity are associated with improved health outcomes, including cancer-related outcomes [9,106–110]. Nutritional intake and physical activity level are each integral to energy balance, where energy intake (diet) that exceeds expenditure (physical activity) over the long term may contribute to obesity and obesity-related cancers [111]. In addition to energy balance, both dietary composition and physical activity are linked to cancer-relevant biological pathways independently of adiposity that are relevant to EC, including insulin sensitivity, dyslipidaemia, oxidative stress, and inflammation [111,112]. Our umbrella review found that women who adhered to a healthy lifestyle had a 46% lower risk of EC compared with those who did not. However, the level of heterogeneity was high. This may be partially explained by differences in population characteristics (e.g., age, race/ethnicity, geography, menopausal status), methods of lifestyle score derivation (e.g., WCRF, ACS, HLI, Health Index), and duration of follow-up (ranging from ~8–18 years). Furthermore, these lifestyle scores vary in both structure and component inclusion, which may contribute to inconsistent effect estimates across studies. For example, the WCRF score typically includes approximately 7 components focused on body weight, diet, and physical activity; the Health Index incorporates around 5 components; the ACS score comprises ~11 components; whereas the HLI is more comprehensive (approximately 20 components) and includes additional behaviours such as smoking alongside diet, physical activity, and alcohol consumption. These differences suggest that the scores do not capture equivalent lifestyle constructs. All 5 individual studies in this meta-analysis were large cohort studies and reported significant inverse associations between adherence to a healthy lifestyle and EC risk. The summary effect estimate was 2.3-fold higher than the largest study’s effect size, suggesting that the summary effect may overestimate the true effect size.

Regarding other outcomes, healthy lifestyle interventional studies have not found evidence of improved QoL in women with EC. Cardiometabolic diseases were reported as possibly the main cause of death in women with EC (35.9%) [113]. However, no significant cardiometabolic events or mortality (all-cause or EC) were reported in the studies investigating these outcomes in our review, possibly due to insufficient follow-up time.

Compared to the epidemiological studies on breast cancer, there is a paucity of evidence on the association between diet and lifestyle quality and EC-related outcomes. Although the results of breast cancer studies have been mixed, a substantial amount of evidence indicates favourable outcomes, such as lower mortality risk from breast-cancer, all-cause and non-breast cancer-related mortality, better quality of life, better mental health, and physical functioning were reported in breast cancer survivors who were following a healthy diet and/ or lifestyle [114–118].

### Strengths and limitations

Our umbrella review has comprehensively assessed all available systematic reviews and meta-analyses on diet quality and lifestyle and several EC outcomes. A robust, systematic search strategy of six electronic databases was conducted. Our selection of studies was based on a pre-specified eligibility criteria that followed PICO elements. Two reviewers, independently at all stages, selected studies and extracted data to ensure that all relevant studies were included. The methodological quality of each included systematic review was assessed using a validated method, AMSTAR 2, by two independent reviewers. We did not limit our umbrella review to meta-analyses only. Systematic reviews without meta-analyses identified a few newly published studies, which we included in our pooled estimates when appropriate. We expanded the study designs of individual studies to include interventional along with observational studies. We re-performed four main analyses on the associations between MDS and EC risk, dietary patterns, and EC risk, combined dietary and lifestyle patterns and EC risk, and lifestyle intervention and QoL. Additionally, we pooled estimates from two RCTs on lifestyle interventions and QoL outcome. Overlapping primary studies were considered in our analysis by quantifying and graphically reporting the level of overlap for each pair of systematic reviews. We rated the strength of evidence for each re-analysed systematic review and meta-analysis. For the association between lifestyle and EC risk, the studies were mostly prospective cohort studies.

Limitations of our review include the small number of individual studies in the re-performed meta-analyses (≤ 5 studies per synthesis) and the limited availability of evidence for post-diagnosis outcomes, including recurrence, overall and EC-specific survival, mortality, and cardiometabolic outcomes. Follow-up durations were generally short, and events were rare in some analyses, which may limit the robustness and interpretability of the findings. Consequently, formal assessments of small-study effects and excess significance bias were not feasible, and 95% prediction intervals could not be estimated.

There was also a high level of overlap in the primary studies across the included reviews, particularly for the associations between MDS and EC risk, lifestyle factors and EC risk, and lifestyle interventions and QoL. To mitigate this, we prioritised estimates from the most recent, comprehensive, or higher-quality meta-analyses. Additionally, differences in effect size metrics across studies (e.g., odds ratios, hazard ratios, and standardised mean differences [Hedges g]) may limit comparability across syntheses.

Heterogeneity in the construction and operationalisation of diet quality and lifestyle indices (e.g., variations in dietary and composite lifestyle scores) may have contributed to between-study variability in effect estimates. Although most primary studies adjusted for key confounders (e.g., age, body mass index, smoking, and family history), residual confounding cannot be excluded. Furthermore, for MDS and dietary patterns and EC risk, the primary studies were predominantly case-control studies and are inherently prone to selection bias. Although no language restrictions were applied during the search, all included reviews were published in English, which may introduce language bias. Finally, the possibility of selective outcome reporting in the included systematic reviews cannot be excluded. Dose-response meta-analyses were not performed in this umbrella review.

## Conclusion

This umbrella review is the first comprehensive synthesis of evidence on adherence to dietary and lifestyle recommendations in relation to EC incidence and health-related outcomes in women diagnosed with EC. Most of the available evidence relates to EC incidence, whereas data on survivorship outcomes remain limited. The findings suggest that healthier dietary and lifestyle patterns are associated with EC incidence; however, the evidence was weak to suggestive and predominantly of moderate to critically low methodological quality.

Given the limited number of studies, this review could not robustly assess whether adherence to a healthy diet and lifestyle is associated with post-diagnosis outcomes, including recurrence, survival, quality of life, or cardiometabolic events. Further observational and interventional studies that account for changes in dietary and lifestyle behaviours over time among EC survivors are needed, as well as larger, higher-quality studies examining both EC incidence and survivorship outcomes.

## Supporting information

S1 File Supplementary materials containing Supplementary Tables S1-S4 and Supplementary Checklist S5. (DOCX)
