# Supplementary material for: Dietary and lifestyle quality and endometrial cancer: Incidence and survivorship outcomes—An umbrella review of systematic reviews and meta-analyses
Source: PLoS One. 2026 Oct 14;21(10):e0360238. doi: 10.1371/journal.pone.0360238 (PMC13667765; doi:10.1371/journal.pone.0360238)
Supplement: Supplementary file 1 [file pone.0360238.s001.docx]

# Supplemental material

## **Supplementary Table S1** Detailed search strategy across 6 databases

| **Database** | **Search terms** |
| --- | --- |
| **PubMed** | ("diet*"[All Fields] OR "diet*"[MeSH Terms] OR "dietary guidelines"[All Fields] OR "diet Mediterranean"[All Fields] OR "diet, Mediterranean"[MeSH Terms] OR "alternate Mediterranean diet"[All Fields] OR "diet, healthy"[MeSH Terms] OR "healthy diet"[All Fields] OR "healthy eating"[All Fields] OR "healthy eating index*"[All Fields] OR "alternative healthy eating index*"[All Fields] OR "Dietary Approaches to Stop Hypertension"[All Fields] OR "dash diet"[All Fields] OR "dietary approaches to stop hypertension"[MeSH Terms] OR "American cancer society"[MeSH Terms] OR "American cancer society"[All Fields] OR "world cancer research fund*"[All Fields] OR "cancer prevention guidelines"[All Fields] OR "Australian Dietary Guidelines"[All Fields] OR "diet therapy"[MeSH Terms] OR "diet modification"[All Fields] OR "dietary modification"[All Fields] OR "dietary intervention"[All Fields] OR "diet intervention"[All Fields] OR "lifestyle*"[All Fields] OR "life style"[MeSH Terms] OR "lifestyle index"[All Fields] OR "lifestyle therapy"[All Fields] OR "lifestyle changes"[All Fields] OR "lifestyle modification"[All Fields] OR "lifestyle intervention"[All Fields])  AND  ("endometrial neoplasms"[MeSH Terms] OR "endometrial neoplasms"[All Fields] OR "endometrial cancer"[All Fields] OR "uterine cancer"[All Fields] OR "uterine neoplasms"[All Fields] OR "uterine neoplasms"[MeSH Terms] OR "endometrial malignancy"[All Fields] OR "endometrial tumor"[All Fields] OR "endometrial tumour"[All Fields])  Filters: Meta-Analysis, Systematic Review |
| **Cochrane library** | #1 ("diet*" OR "diet therapy" OR "Dietary Approaches To Stop Hypertension" OR "dash diet" OR "diet, healthy" OR "diet modification " OR "dietary modification" OR "dietary intervention" OR "diet intervention" OR "diet score" OR "diet index" OR "diet, Mediterranean" OR "alternate Mediterranean diet" OR "American Cancer Society" OR "World cancer research fund" OR "dietary guidelines" OR "diet quality" OR "healthy eating index*" OR "alternative healthy eating index*" OR "Australian Dietary Guidelines" OR "Lifestyle index" OR "Lifestyle change" OR "Lifestyle*" OR "Healthy Lifestyle" OR "Lifestyle intervention" OR "Lifestyle modification" OR "cancer prevention guidelines" ):ti,ab,kw (Word variations have been searched)  #2 MeSH descriptor: [Dietary Approaches To Stop Hypertension] explode all trees  #3 MeSH descriptor: [Diet Therapy] explode all trees  #4 MeSH descriptor: [Diet, Healthy] explode all trees  #5 MeSH descriptor: [Diet, Mediterranean] explode all trees  #6 MeSH descriptor: [American Cancer Society] explode all trees  #7 MeSH descriptor: [Nutrition Policy] explode all trees  #8 MeSH descriptor: [Feeding Behavior] explode all trees  #9 MeSH descriptor: [Healthy Lifestyle] explode all trees  #10 MeSH descriptor: [Life Style] explode all trees  #11 #1 OR #2 OR #3 OR #4 OR #5 OR #6 OR #7 OR #8 OR #9 OR #10  #12 ("endometrial neoplasms" OR "endometrial cancer" OR "endometrial carcinoma" OR "endometrial malignancy" OR "endometrial tumour" OR "uterine tumour" OR "uterine cancer" OR "uterine carcinoma" OR "uterine neoplasms" OR "uterine malignancy"):ti,ab,kw (Word variations have been searched)  #13 MeSH descriptor: [Endometrial Neoplasms] explode all trees  #14 MeSH descriptor: [Uterine Neoplasms] explode all trees  #15 #12 OR #13 OR #14  #16 #11 AND #15 |
| **EMBASE** | 'diet*' OR 'diet'/exp OR 'diet quality' OR 'dietary change'/exp OR 'dietary change' OR 'diet intervention' OR 'dietary intervention' OR 'diet modification' OR 'dietary modification' OR 'diet therapy' OR 'diet therapy'/exp OR 'dietary guidelines' OR 'healthy eating index'/exp OR 'alternative healthy eating index'/exp OR 'alternative healthy eating index' OR 'mediterranean diet'/exp OR 'mediterranean diet' OR 'alternate mediterranean diet'/exp OR 'alternate mediterranean diet' OR 'dash diet'/exp OR 'dash diet' OR 'australian dietary guidelines' OR 'american cancer society'/exp OR 'american cancer society' OR 'world cancer research fund' OR 'cancer prevention guidelines' OR 'lifestyle'/exp OR 'lifestyle*' OR 'lifestyle modification'/exp OR 'lifestyle modification' OR 'lifestyle intervention'/exp OR 'lifestyle intervention' OR 'lifestyle change' OR 'healthy lifestyle' OR 'healthy lifestyle'/exp  AND  'uterus carcinoma'/exp OR 'uterus carcinoma' OR 'uterine neoplasm' OR 'uterine neoplasms'/exp OR 'uterine neoplasms' OR 'uterus cancer'/exp OR 'uterus cancer' OR 'uterus tumor'/exp OR 'uterus tumor' OR 'uterine malignancy' OR 'endometrial carcinomas' OR 'endometrium carcinoma'/exp OR 'endometrium carcinoma' OR 'endometrial neoplasm' OR 'endometrial neoplasms'/exp OR 'endometrial neoplasms' OR 'endometrium tumor'/exp OR 'endometrium tumor' OR 'endometrial malignancy'  AND  'systematic review'/exp OR 'systematic review' OR 'systematic review (topic)'/exp OR 'systematic review (topic)' OR 'meta analysis'/exp OR 'meta analysis' OR 'meta-analysis' OR 'meta analysis (topic)'/exp |
| **SCOPUS** | ( TITLE-ABS-KEY ( diet* ) )  OR  ( TITLE-ABS-KEY ( diet  AND quality ) )  OR  ( TITLE-ABS-KEY ( dietary  AND change ) )  OR  ( TITLE-ABS-KEY ( dietary  AND guidelines ) )  OR  ( TITLE-ABS-KEY ( diet  AND index ) )  OR  ( TITLE-ABS-KEY ( diet  AND score ) )  OR  ( TITLE-ABS-KEY ( diet  AND therapy ) )  OR  ( TITLE-ABS-KEY ( diet  AND intervention ) )  OR  ( TITLE-ABS-KEY ( dietary  AND intervention ) )  OR  ( TITLE-ABS-KEY ( diet  AND modification ) )  OR  ( TITLE-ABS-KEY ( dietary  AND modification ) )  OR  ( TITLE-ABS-KEY ( healthy  AND diet ) )  OR  ( TITLE-ABS-KEY ( healthy  AND eating  AND index ) )  OR  ( TITLE-ABS-KEY ( alternative  AND healthy  AND eating  AND index ) )  OR  ( TITLE-ABS-KEY ( australian  AND dietary  AND guidelines ) )  OR  ( TITLE-ABS-KEY ( dash  AND diet ) )  OR  ( TITLE-ABS-KEY ( mediterranean  AND diet ) )  OR (TITLE-ABS-KEY ( 'alternate AND mediterranean AND diet' ) ) OR ( TITLE-ABS-KEY ( american  AND cancer  AND society ) )  OR  ( TITLE-ABS-KEY ( world  AND cancer  AND research  AND fund ) )  OR  ( TITLE-ABS-KEY ( cancer  AND prevention  AND guideline ) )  OR  ( TITLE-ABS-KEY ( lifestyle* ) )  OR  ( TITLE-ABS-KEY ( lifestyle  AND change ) )  OR  ( TITLE-ABS-KEY ( healthy  AND lifestyle ) )  OR  ( TITLE-ABS-KEY ( 'lifestyle  AND modification' ) )  OR  ( TITLE-ABS-KEY ( lifestyle  AND intervention ) )  AND  ( TITLE-ABS-KEY ( endometrial  AND  cancer ) )  OR  ( TITLE-ABS-KEY ( endometrial  AND  neoplasm ) )  OR  ( TITLE-ABS-KEY ( endometrial  AND  carcinoma ) )  OR  ( TITLE-ABS-KEY ( endometrial  AND  tumor ) )  OR  ( TITLE-ABS-KEY ( endometrial  AND  tumour ) )  OR  ( TITLE-ABS-KEY ( endometrial  AND  malignancy ) )  OR  ( TITLE-ABS-KEY ( uterine  AND  tumor ) )  OR  ( TITLE-ABS-KEY ( uterine  AND  tumour ) )  OR  ( TITLE-ABS-KEY ( uterine  AND  carcinoma ) )  OR  ( TITLE-ABS-KEY ( uterine  AND  cancer ) )  OR  ( TITLE-ABS-KEY ( uterine  AND  neoplasm ) )  OR  ( TITLE-ABS-KEY ( uterine  AND  malignancy ) )  AND  ( TITLE-ABS-KEY ( systematic AND reviews ) ) OR ( TITLE-ABS-KEY ( meta AND analysis ) ) OR ( TITLE-ABS-KEY ( meta-analysis ) ) |
| **Web of Science** | (((((((((((((((((((((((((TS=(diet*)) OR TS=("diet quality")) OR TS=("dietary guidelines" )) OR TS=("diet index")) OR TS=("diet score")) OR TS=("healthy diet")) OR TS=("dietary change")) OR TS=("diet therapy" )) OR TS=("diet intervention")) OR TS=("dietary intervention")) OR TS=("diet modification")) OR TS=("dietary modification")) OR TS=("healthy eating index")) OR TS=("alternative healthy eating index")) OR TS=("Mediterranean diet")) OR TS=("alternate Mediterranean diet")) OR TS=("dash diet")) OR TS=("Australian dietary guidelines")) OR TS=("American cancer society")) OR TS=("world cancer research fund")) OR TS=("cancer prevention guidelines")) OR TS=(lifestyle*)) OR TS=("lifestyle modification")) OR TS=("lifestyle intervention")) OR TS=("lifestyle change")) OR TS=("healthy lifestyle")  AND  (((((((((((TS=("endometrial cancer")) OR TS=("endometrial neoplasms")) OR TS=("endometrial carcinoma")) OR TS=(endometrial tumor)) OR TS=(endometrial tumour)) OR TS=("endometrial malignancy")) OR TS=("uterine cancer")) OR TS=("uterine neoplasms")) OR TS=("uterine carcinoma")) OR TS=("uterine tumor")) OR TS=("uterine tumour")) OR TS=("uterine malignancy")  AND  (TS=(systematic reviews)) OR TS=(meta analysis) |
| **CINAHL** | diet* OR diet quality OR dietary guidelines OR dietary patterns OR dietary habits OR dietary changes OR healthy diet OR diet therapy OR dietary intervention OR dietary modification OR diet score OR diet index OR diet and exercise interventions OR dash diet or dietary approaches to stop hypertension OR mediterranean diet OR alternate mediterranean diet OR healthy eating index OR alternative healthy eating index OR australian dietary guidelines OR american cancer society OR world cancer research fund OR cancer prevention guidelines OR lifestyle* OR lifestyle index OR lifestyle modification OR lifestyle change OR lifestyle intervention OR healthy lifestyle OR lifestyle therapy  AND endometrial neoplasms OR endometrial cancer OR endometrial carcinoma OR endometrial malignancy OR endometrial tumor OR endometrial tumour OR uterine cancer OR uterine neoplasms OR uterine carcinoma OR uterine tumor OR uterine tumour OR uterine malignancy  AND  PT systematic review or meta-analysis  Expanders - Apply equivalent subjects  Search modes - Boolean/Phrase |

## **Supplementary Table S2** Summary of systematic reviews/meta-analyses on diet/lifestyle quality and endometrial cancer (N=21)

| **Author, year** | **Study type** | **No of**  **studies^a b^ and study designs** | **Countries** | **No of participants,**  **No of cases** | **Median follow-up*** | **Age** | **Exposure/**  **intervention** | **Outcomes** | **Main findings**  **RR/OR/HR/MD (95% CI)** | **Quality** |
| --- | --- | --- | --- | --- | --- | --- | --- | --- | --- | --- |
| **Dietary indices/patterns** |  |  |  |  |  |  |  |  |  |  |
| Zhang,  2021^a^ | SR and MA | 3 cohort, 1 case-cohort, 3 case-control | USA, Canada, Italy | N=244,045  Cases= 5,222 | 9.5 to 17.9 years | 40 to 79 years | HEI-2005, HEI-2010, AHEI-2010, MDS, aMDS, DASH, diet score, recommended food score | EC risk | Diet Score, RR=0.81 (0.67 to 0.98).  Healthy Diet Score RR= 0.91 (0.53 to 1.56)  HEI-2010 RR= 1.11 (0.93 to 1.33)  AHEI -2010 RR= 0.98 (0.82 to 1.17)  aMDS, RR= 0.98 (0.82 to 1.17)  DASH, RR= 1.00 (0.84 to 1.19)  Recommended Foods Score, RR= 0.87 (0.61 to 1.22)  MDS, RR= 0.51 (0.28 to 0.92)  MDS, RR= 0.43 (0.34 to 0.56)  HEI-2005 RR= 0.83 (0.52 to 1.34)  Pooled MDS RR for one unit increment of the MDS= 0.87 (0.81 to 0.93), | Moderate |
| Si C J,  2017 ^b^ | SR and MA | 1 cohort, 4 case-control | USA, Canada, Italy | N= 79,762  Cases= 2,617 | 16.1 years | ≤ 85 years | Dietary patterns | EC risk | Original pooled OR of EC risk for highest vs lowest categories of:  - Healthy dietary pattern (4 cohort) = 0.74 (0.62 to 0.88), I2= 72%, p< 0.0001)  - Western dietary pattern (3 cohort studies) = 1.37 (1.15 to 1.64), I2= 81%, p < 0.00001  Re analysis of pooled estimates excluding studies with single food groups as exposures:  - Healthy dietary pattern (3 studies with complete data) OR=0.89 (0.76 to 1.05), I^2^= 0%,  - Western dietary pattern (3 studies with complete data) OR=1.20 (1.03 to 1.39), I^2^= 0%, | Low |
| Alizadeh,  2020^a^ | SR and MA | 1 cohort, 4 case-control | USA, Canada, Italy | N= 79,762  Cases= 2,617 | 16.1 years | ≤ 85 years | Healthy vs western dietary patterns | EC risk | Pooled OR of EC risk for highest vs lowest categories of:  - Healthy dietary patterns, OR=0.84 (0.72 to 0.98), I^2^ = 47.2%, P= 0.108  - Western dietary pattern, OR=1.19 (1.01 to 1.41), I^2^ = 47.2%, P= 0.35 | Moderate |
| Grosso G,  2017^a^ | SR and MA | 1 cohort, 4 case-control | USA, Canada, Italy | N= 79,762  Cases= 2,617 | 16.1 years | ≤ 85 years | Dietary patterns | Cancer risk | Pooled OR of EC risk for highest vs lowest categories of:  - Healthy dietary patterns, RR= 0.83 (0.67 to 1.04), I^2^=32% for 6 datasets of case-control studies and RR= 0.96 (0.82 to 1.12), I^2^= 0% for 2 datasets of cohort studies.  - Western dietary patterns, RR=1.19 (0.99 to 1.43), I^2^=44% for 8 datasets of case-control studies and RR=1.10 (0.94 to 1.29), I^2^= 0% for 3 datasets of cohort studies. | Low |
| Verberne L, 2010^a^ | SR | 1 case-control | USA | N=1280 Cases=647 | NA | 35 to  79  years | MDS | EC risk | EC risk for highest vs lowest categories of MDS, OR=0.92 (0.59 to 1.43) | Critically low |
| Schwinshackle L, 2015^a^ | SR and MA | 1 cohort, 2 case-control | USA, Italy | N=90,774  Cases= 3,450 | 13.3 years* | 35 to  79  years | MDS | Cancer risk,  All-cause or cancer specific mortality  Cancer recurrence | Pooled OR of 3 studies =0.72 (0.40 to 1.31), I^2^=94%  For each study Q5 vs Q1 and EC:  - George et al, HR= 0.98 (0.82 to 1.17)  - Filomeno et al, OR= 0.43 (0.34 to 0.56)  - Dalvi et al, OR=0.92 (0.59 to 1.43) | Low |
| Morze,  2021^a^ | SR and MA | 1 cohort, 3 case-control | USA, Italy | N= 91,047  Cases= 3,588 | 13.3 years* | 35 to  79  years | MDS | Cancer risk, recurrence,  all-cause or cancer specific mortality | Pooled effect of EC risk for highest vs lowest categories of MDS:  - RR=0.67 (0.41 to 1.11), I^2^ = 91% (4 studies)  - OR=0.58 (0.35 to 0.95), I^2^ = 77% (3 case-control studies)  - RR=0.98 (0.82 to1.17) (1 cohort) | High |
| MazandaranianM R, 2019^a^ | SR | 1 cohort, 2 case-control | USA, Italy | N= 85,968  Cases= 2,177 | 13.3 years* | 35 to  79  years | MDS | Cancer risk | For each study Q5 vs Q1 and EC:  - George et al, HR= 0.98 (0.82 to 1.17)  - Dalvi et al, OR=0.92 (0.59 to 1.43)  - Ricceri et al, OR=0.51 (0.28 to 0.92) | Critically low |
| Mohsenpour M A, 2019^a^ | SR and MA | 1 cohort | USA | N=84,415 Cases=1,392 | 13.3 years* | 50 to 79 years | DASH | EC risk | For highest vs lowest score and EC risk  - HEI-2010, RR= 1.11 (0.93 to 1.33)  - AHEI-2010, RR= 0.98 (0.82 to 1.17)  - aMDS, RR= 0.98 (0.82 to 1.17)  - DASH, RR= 1.00 (0.84 to 1.19) | High |
| **Lifestyle indices/patterns** |  |  |  |  |  |  |  |  |  |  |
| Fiona C. Malconson, 2023^a^ | SR and MA | 1 multicentric case-control | Italy | N= 1,362  Cases= 454 | - | Cases (median age=60 years, range 18–79)  Controls=  (median age=61 years, range 19–79) | WCRF | EC risk | Highest compared with the lowest WCRF score quartile   - OR = 0·42, (0·30 to 0·61)   Highest compared with the lowest WCRF (only dietary recommendations)  score tertile   - OR = 0·67 (0·46 to 0·96) |  |
| Hui Sun,  2021 | SR and MA | 4 cohort, 1 case-cohort | USA, Canada, 9 European countries | N= 723,304  Cases= 4,548 | 8 to 17.9 years | 25 to  79  years | WCRF  ACS  HLI  Health index | EC risk | Pooled HR of EC risk for highest vs lowest categories of healthy lifestyle, HR= 0.54 (0.40 to 0.73), I^2^= 86.1%, p<0.01 | High |
| Zhang Y B, 2020 | SR and MA | 4 cohort, 1 case-cohort | USA, Canada, 9 European countries | N= 723,304  Cases= 4,548 | 8 to 17.9 years | 25 to  79  years | WCRF  ACS  HLI  Health index | Cancer risk or mortality | Pooled HR of EC risk for highest vs lowest categories of healthy lifestyle, HR= 0.54 (0.40 to 0.73), I^2^= 86.1%, p<0.01 | High |
| M Solan,  2020^a^ | SR and MA | 1 cohort | 9 European countries | N=386,355  Cases=1148 | 11 years | 25 to 70 years | WCRF | Any health outcome | High adherence associated significantly with lower risk of EC, HR=0.77 (0.62 to 0.94) | Critically low |
| Kohler L N, 2016^a^ | SR | 3 cohort | USA, 9 European countries | N= 928,589  Cases= 3175 | 11 to 13.6 years  Mean 12.6 years (1 cohort) | 25 to  79  years | WCRF  ACS | Cancer risk or mortality | - Thomson, 2014, no significant association between highest ACS scores vs lowest and risk of EC, HR=0.73 (0.49 to 1.09). Higher ACS scores were not associated with lower risk of death from endometrial 0.59 (0.26 to 1.37)  - Kabat, 2015, high adherence to ACS associated significantly with lower risk of EC, HR=0.40 (0.34 to 0.46), p<0.0001 (cancer mortality and overall mortality were reported for all cancers collectively)  - Romaguera, 2012, high adherence to WCRF was associated significantly with lower risk of EC, HR=0.77 (0.62 to 0.94)  No reported mortality outcomes for EC | Moderate |
| Yeganeh L, 2018^a^ | SR and MA | 2 RCT | USA | LI= 64  UC= 56 | Baseline, 3, 6 and 12 months * | Mean age 54-57 (LI)  and  55.5-58.9 (UC) | LI= 6 months behavioural PA and nutritional counselling  UC= Information handouts | Primary:  Cancer recurrence  and  Overall survival  Secondary:  QoL | Pooled MD of the effect of LI vs UC on global and domain specific QoL at 3- and 6-months follow up showed no significant difference between the groups.  No reported primary outcomes for EC | Critically low |
| Agnew H, 2023^b^ | SR and MA | 12 RCT | USA, Australia and New Zealand | LI= 300  UC= 256 | Baseline, 3, 6, 12, 24 months | Mean age 53- 66 years | LI= 6 months behavioural PA and nutritional counselling, restricted calories, Moderate PA  UC= Information handouts | Primary: overall survival, and  frequency of adverse events  Secondary:  Recurrence, cancer specific survival,  weight loss,  cardiovascular or metabolic events  and  QoL | LI and overall survival at 3, 6, 12 and 24 months: Insufficient data for HR, so RR was used instead:   - No evidence of association at 3 months (Clark 2021, Edbrooke 2022), 6 months (McCarroll 2014, Allison 2016, Janda 2021, Maxwell-Smith 2019, Yeh 2021) and at 12 months (McCarroll 2014, Zamorano 2021) (No deaths reported). - No association found at 24 months RR(mortality)= 0.23 (0.01 to 4.55), p= 0.34 (Von Gruenigen 2008)   No evidence on cancer specific survival at 3, 6, 12, and 24 months (no cancer specific deaths reported for the two studies reporting this outcome)  LI and QoL at 9 weeks, 3, 6 and 12 months:   - No improvement in QoL (FACT-G) at 9 weeks MD=3.51 (10.96 to 17.98) P= 0.63 (Edbrooke 2022) - No improvement in QoL (FACT-G) at 3 months MD=5.75 (-11.26 to 22.76) P= 0.53 (Edbrooke 2022) - No improvement in QoL (SF-12 Physical/Mental Health questionnaire) at 3 months. MD could not be calculated (Cohen 2018) - No improvement in QoL at 6 months (FACT-G questionnaire): Pooled MD= 2.51 (-5.61 to 10.64), P= 0.54, I^2^= 83% (McCarroll 2014 and von Gruenigen 2008) - No improvement in QoL (SF-12 Physical Health questionnaire) at 6 months: MD= -2.29 (-7.34 to 2.76) P= 0.37 (Allison 2016) - No improvement in QoL at 12 months (FACT-G questionnaire): MD=2.77 (-0.65 to 6.20) P= 0.11, I^2^=0% (McCarroll 2014 and von Gruenigen 2008) - No improvement in QoL at 12 months (SF-12 Physical Health questionnaire): MD=-3.50 (-8.85 to 1.85) P= 0.20 (Zamorano 2021) - No improvement in QoL at 12 months (SF-12 Mental Health questionnaire): MD=-3.00 (-2.49 to 8.49) P= 0.28 (Zamorano 2021) - No improvement in QoL at 12 months (9-item Patient Health questionnaire): MD=0.90 (-1.79 to 3.59) P= 0.51 (Zamorano 2021)   A subgroup analysis by BMI, showed no difference in QoL (FACT-G) response to the intervention  No recurrence-free survival outcome as no death reported in the one study that reported this outcome.  No significant cardiometabolic events reported (only one episode of congestive heart failure in one RCT at 6 months RR=3.47, (0.15 to 82.21), P = 0.44, other 4 RCTs with 0 events) | High |
| Koutoukidis D A, 2015^b^ | SR | 2 RCT | USA | LI= 64  UC= 56 | Baseline, 3, 6 and 12 months * | Mean age 54-57 (LI)  and  55.5-58.9 (UC) | LI= 6 months behavioural PA and nutritional counselling  UC= Information handouts | QoL | Von Gruenigen 2009,  Significant improvement in self-efficacy related to social pressure (p=0.03) and restraint (p=0.02) in LI compared to UC group. Significant improvement in weight, physical activity and nutrient intake in LI compared to UC group. No difference in QoL, fatigue and depression between the groups.  McCarroll 2014,  Significant improvement in fatigue at 3 months, physical functioning at 6 months, and self-efficacy at 12 months in LI compared to UC group. Significant improvement in overall QoL over time (3&6 months) in LI group. | Critically low |
| Buchanan A, 2020 | SR | 4 RCT | USA, UK | LI= 118  UC= 102 | Baseline, 2, 3, 6 and 12 months * | Mean age 54-62.1 (LI)  and  55.5-62.1 (UC) | LI= 6 months behavioural PA and nutritional counselling/  Manual, group-based sessions, phone sessions or texts (caloric restriction and ↑PA)  UC= Information handouts | QoL | McCarroll 2014,  Improvements in domain specific QoL (Fatigue domain at 3months and physical well-being domain at 6 months in LI compared to UC group from baseline (p=0.008 and P=0.48 respectively). Significant increase in total FACT-G scores within LI group at 3 months 87.1 (SD 7.6), p=0.008 and 6 months 86.6 (SD 9.3), p=0.0005 from baseline.  Von Gruenigen 2009,  Significant improvement in self-efficacy related to social pressure (p=0.03) and restraint (p=0.02) in LI compared to UC group. Significant improvement in weight, physical activity and nutrient intake in LI compared to UC group. No difference in QoL, fatigue and depression between the groups.  Haggerty 2017,  Improvement in Physical health component (SF-12) in telemedicine group compared to text4diet P=0.04  Koutoukidis, 2019,  Global QoL improved in LI group at 6 months; MD= 8.9 (0.9 to 16.8), P=0.029. | Low |
| Burden S, 2019 | SR | 1 RCT | USA | LI= 41  UC= 34 | Baseline, 3, 6 and 12 months * | Mean age 57 (LI)  and  58.9 (UC) | LI= 6 months behavioural PA and nutritional counselling  UC= Information handouts | Primary:  Overall survival,  Secondary/other cancers incidence, morbidities  Secondary:  Dietary change, or change in body composition | LI group had an increased LSI score compared to the UC group at 3, 6 and 12 months from baseline [MD between groups=8.7 (4.2 to 13.2) at 3 months, MD=11.2 (7.2 to 15.2) at 6 months and MD=7.8 (4.2 to 11.4) at 12 months, p<0.001 at all timepoints].  No outcomes on survival, secondary cancers, comorbidities reported | High |
| Chlebowski, 2016^a b^ | SR | 2 RCT | USA | LI=  UC= | Baseline, 3, 6 and 12 months * | Mean age  57-59.4 (LI)  and  58.9-59.4  (UC) | LI= 6 months behavioural PA and nutritional counselling/  Manual, group-based sessions, phone sessions or texts (caloric restriction and ↑PA)  UC= Information handouts | Weight loss and cancer end points | McCarroll 2014,  There was significant association between ↓BMI and ↑in WEL scores in all domains (total, physical, social, positive, availability) except for negative emotions domain.  - QoL (FACT-G): Fatigue domain at 3months and physical well-being domain at 6 months were significantly difference between the groups from baseline (p=0.008 and P=0.48 respectively). Significant increase in total FACT-G scores within LI group at 3 months 87.1 (SD 7.6), p=0.008 and 6 months 86.6 (SD 9.3), p=0.0005. No association between BMI loss and QoL. | Critically low |
| Smits A,  2015^a b^ | SR and MA | 2 RCT | USA | LI= 64  UC= 56 | Baseline, 3, 6 and 12 months * | Mean age 54-57 (LI)  and  55.5-58.9 (UC) | LI= 6 months behavioural PA and nutritional counselling  UC= Information handouts | QoL | -McCarroll 2014,  Significant improvement in physical functioning at 6 months, fatigue at 3 months, and self-efficacy at 12 months in LI compared to UC group. Significant improvement in overall QoL over time (3&6 months) in LI group.  -Von Gruenigen 2009,  Significant improvement in self-efficacy and social pressure in LI compared to UC group. Significant improvement in weight, physical activity and nutrient intake in LI compared to UC group. No difference in QoL, fatigue and depression between groups. | Critically low |

Abbreviations: SR, systematic review; MA, meta-analysis; EC, endometrial cancer; RR, relative risk; OR, Odds ratio; HR, hazard ratio; MD, mean difference; CI, confidence interval, p, p-value; I^2^,percentage of heterogeneity; HEI, Healthy Eating Index; AHEI, Alternative Healthy Eating Index; MDS, Mediterranean Diet Score; aMDS, alternate Mediterranean Diet Score; DASH, Dietary Approach to Stop Hypertension; WCRF, World cancer research fund; ACS, American cancer society; HLI, Healthy Lifestyle Index; RCTs, randomized controlled trials; QoL, quality of life; LI, lifestyle intervention; UC, usual care; FACT-G, Functional Assessment for Cancer Therapy–General; FACT-F, Functional Assessment of Cancer Therapy —Fatigue; LSI, Leisure score index; PA, physical activity; SF-36, Short-Form 36-Item Health Survey; WEL, Self-efficacy

a Only studies on EC were included

b Only primary studies that meet eligibility criteria were included

## **Supplementary Table S3** AMSTAR 2 quality assessment rating for each included systematic review

|  | Kohler, 2016 | Sun H, 2021 | Zhang, 2021 | Alzeidah, 2020 | Zhang, 2020 | M Solan, 2020 | Smits, 2015 | Grosso, 2017 | Si C J, 2017 | Ali Mohsenpour, 2019 | Buchanan, 2021 | Burden, 2019 | Koutoukidis, 2015 | Morze, 2021 | Chlebowski, 2016 | Verberne, 2010 | Yeganeh, 2018 | Agnew H, 2023 | Schwingshackl, 2015 | Mazandaranian, 2019 | Malconson F. C,2023 |
| --- | --- | --- | --- | --- | --- | --- | --- | --- | --- | --- | --- | --- | --- | --- | --- | --- | --- | --- | --- | --- | --- |
| (1) The research question includes PICO | 1 | 1 | 1 | 1 | 1 | 1 | 1 | 1 | 1 | 1 | 1 | 1 | 1 | 1 | 1 | 1 | 1 | 1 | 1 | 1 | 1 |
| (2) Methods established prior to conducting the review | 1 | 1 | 1 | 1 | 1 | 0 | 1 | 1 | 0 | 1 | 0 | 1 | 0 | 1 | 0 | 0 | 1 | 1 | 1 | 1 | 1 |
| (3) Explained selection of study designs for inclusion | 1 | 1 | 0 | 0 | 1 | 0 | 0 | 0 | 0 | 1 | 1 | 1 | 0 | 1 | 1 | 0 | 1 | 1 | 1 | 0 | 1 |
| (4) A comprehensive literature search strategy used | 1 | 1 | 1 | 1 | 1 | 1 | 1 | 1 | 1 | 1 | 0.5 | 1 | 1 | 1 | 1 | 0.5 | 1 | 1 | 1 | 1 | 1 |
| (5) Study selection by two interviewers | 1 | 1 | 1 | 0 | 1 | 0 | 1 | 0 | 1 | 1 | 1 | 1 | 0 | 1 | 1 | 0 | 0 | 1 | 1 | 1 | 1 |
| (6) Data extracted by two interviewers | 1 | 1 | 1 | 1 | 1 | 1 | 0 | 1 | 0 | 1 | 1 | 1 | 0 | 1 | 1 | 0 | 1 | 1 | 0 | 1 | 1 |
| (7) Excluded studies (list and justification) | 1 | 1 | 1 | 1 | 1 | 0 | 0 | 1 | 1 | 1 | 1 | 1 | 0 | 1 | 0 | 0 | 0 | 1 | 0 | 0 | 0.5 |
| (8) The included studies described in adequate detail | 1 | 1 | 1 | 1 | 1 | 1 | 1 | 1 | 0.5 | 1 | 1 | 1 | 1 | 1 | 1 | 1 | 1 | 1 | 1 | 1 | 1 |
| (9) A satisfactory technique for RoB assessment of included studies | 1 | 1 | 1 | 1 | 1 | 0 | 1 | 0 | 1 | 1 | 1 | 1 | 1 | 1 | 1 | 0 | 1 | 1 | 1 | 0 | 1 |
| (10) Sources of funding for included studies was reported | 0 | 0 | 0 | 0 | 0 | 0 | 0 | 0 | 0 | 1 | 0 | 1 | 0 | 0 | 0 | 0 | 0 | 1 | 0 | 0 | 0 |
| (11) Statistical combination of results was appropriate (MA) | NA | 1 | 1 | 1 | 1 | 1 | 1 | 1 | 0 | 1 | NA | 1 | NA | 1 | NA | NA | 1 | 1 | 1 | NA | 1 |
| (12) Impact of RoB in individual studies was assessed (MA) | NA | 1 | 1 | 1 | 1 | 1 | 1 | 0 | 1 | 1 | NA | 1 | NA | 1 | NA | NA | 1 | 1 | 1 | NA | 1 |
| (13) Accounted for RoB in individual studies | 1 | 1 | 1 | 1 | 1 | 1 | 1 | 1 | 1 | 1 | 1 | 1 | 1 | 1 | 1 | 1 | 1 | 1 | 1 | 1 | 1 |
| (14) Any heterogeneity was explained and discussed | 1 | 1 | 1 | 1 | 1 | 1 | 1 | 1 | 1 | 1 | 1 | 1 | 1 | 1 | 1 | 1 | 1 | 1 | 1 | 0 | 1 |
| (15) Publication (small study) bias and likely impact was assessed (MA) | NA | 1 | 1 | 1 | 1 | 1 | 0 | 1 | 1 | 1 | NA | 1 | NA | 1 | NA | NA | 0 | 1 | 1 | NA | 1 |
| (16) Conflict of interest and any funding to conduct the review | 1 | 1 | 1 | 1 | 1 | 1 | 1 | 1 | 1 | 1 | 1 | 1 | 1 | 1 | 1 | 1 | 1 | 1 | 1 | 1 | 1 |
| Total quality rating | M | H | M | M | H | CL | CL | L | L | H | L | H | CL | H | CL | CL | CL | H | L | CL | H |

Abbreviations: PICO Population Intervention Comparator Outcome, RoB Risk of Bias, MA Meta-analysis. Score allocation: Yes= 1, Partial yes=0.5, No=0, Not applicable= NA, Total quality rating: H=High, M=Moderate, L=Low, CL=Critically Low

## **Supplementary Table S4** Citation matrix of overlap in primary studies across the included systematic reviews

|  | Zhang, 2021 | Verberne, 2010 | Schwinshackle, 2015 | Morze, 2021 | Mazandaranian, 2019 | Mohsenpour, 2019 | Si C J, 2017 | Alizadeh, 2020 | Grosso, 2017 | Kohler, 2016 | M Solan, 2020 | Hui Sun, 2021 | Zhang Y B, 2020 | Yeganeh, 2018 | Koutoukidis, 2015 | Agnew H, 2023 | Buchanan, 2020 | Chlebowski, 2016 | Burden, 2019 | Smits, 2015 | Malconson F. C,2023 |
| --- | --- | --- | --- | --- | --- | --- | --- | --- | --- | --- | --- | --- | --- | --- | --- | --- | --- | --- | --- | --- | --- |
| Arthur 2019 | x |  |  |  |  |  |  |  |  |  |  | x | x |  |  |  |  |  |  |  |  |
| George 2015 | x |  | x | x | x | x |  |  |  |  |  |  |  |  |  |  |  |  |  |  |  |
| Mai 2005 | x |  |  |  |  |  |  |  |  |  |  |  |  |  |  |  |  |  |  |  |  |
| Arthur 2018 | x |  |  |  |  |  |  |  |  |  |  | x | x |  |  |  |  |  |  |  |  |
| Ricceri 2017 | x |  |  | x | x |  |  |  |  |  |  |  |  |  |  |  |  |  |  |  |  |
| Filomeno 2015 | x |  | x | x |  |  |  |  |  |  |  |  |  |  |  |  |  |  |  |  |  |
| Chandran 2010 | x |  |  |  |  |  |  |  |  |  |  |  |  |  |  |  |  |  |  |  |  |
| Dalvi 2007 |  | x | x | x | x |  | x | x | x |  |  |  |  |  |  |  |  |  |  |  |  |
| Canchola 2015 |  |  |  |  |  |  | x | x | x |  |  |  |  |  |  |  |  |  |  |  |  |
| McCann 2001 |  |  |  |  |  |  | x | x | x |  |  |  |  |  |  |  |  |  |  |  |  |
| Biel 2011 |  |  |  |  |  |  | x | x | x |  |  |  |  |  |  |  |  |  |  |  |  |
| Bravi 2015 |  |  |  |  |  |  | x | x | x |  |  |  |  |  |  |  |  |  |  |  |  |
| Thomson 2014 |  |  |  |  |  |  |  |  |  | x |  |  |  |  |  |  |  |  |  |  |  |
| Kabat 2015 |  |  |  |  |  |  |  |  |  | x |  | x | x |  |  |  |  |  |  |  |  |
| Romaguera 2012 |  |  |  |  |  |  |  |  |  | x | x | x | x |  |  |  |  |  |  |  |  |
| Dartois 2014 |  |  |  |  |  |  |  |  |  |  |  | x | x |  |  |  |  |  |  |  |  |
| Blanchard 2008 |  |  |  |  |  |  |  |  |  |  |  |  |  |  | x |  |  |  |  |  |  |
| Von Gruenigen 2009/Von Von Gruenigen 2008 |  |  |  |  |  |  |  |  |  |  |  |  |  | x | x | x | x |  |  | x |  |
| McCarroll 2014/ Von Gruenigen 2012 |  |  |  |  |  |  |  |  |  |  |  |  |  | x | x | x | x | x | x | x |  |
| Allison 2016/ Haggerty 2017 |  |  |  |  |  |  |  |  |  |  |  |  |  |  |  | x | x |  |  |  |  |
| Koutoukidis 2019 |  |  |  |  |  |  |  |  |  |  |  |  |  |  |  |  | x |  |  |  |  |
| Edbrooke 2022 |  |  |  |  |  |  |  |  |  |  |  |  |  |  |  | x |  |  |  |  |  |
| Zamorano 2021 |  |  |  |  |  |  |  |  |  |  |  |  |  |  |  | x |  |  |  |  |  |
| Cohen 18 |  |  |  |  |  |  |  |  |  |  |  |  |  |  |  | x |  |  |  |  |  |
| Clark 2021 |  |  |  |  |  |  |  |  |  |  |  |  |  |  |  | x |  |  |  |  |  |
| Janda 2021 |  |  |  |  |  |  |  |  |  |  |  |  |  |  |  | x |  |  |  |  |  |
| Maxwell‐Smith 2019 |  |  |  |  |  |  |  |  |  |  |  |  |  |  |  | x |  |  |  |  |  |
| Mohammad 2019 |  |  |  |  |  |  |  |  |  |  |  |  |  |  |  | x |  |  |  |  |  |
| Yeh 2021 |  |  |  |  |  |  |  |  |  |  |  |  |  |  |  | x |  |  |  |  |  |
| Esposito G, 2023 |  |  |  |  |  |  |  |  |  |  |  |  |  |  |  |  |  |  |  |  | x |

Columns: Systematic reviews, Rows: Primary studies (Primary studies that meet our inclusion criteria only were included)

## **Supplementary Checklist S5** PRISMA 2020 checklist

Locations refer to page numbers in the revised manuscript.

| **Section and Topic** | **Item #** | **Checklist item** | **Location where item is reported** |
| --- | --- | --- | --- |
| **TITLE** | | |  |
| Title | 1 | Identify the report as a systematic review. | 1 |
| **ABSTRACT** | | |  |
| Abstract | 2 | See the PRISMA 2020 for Abstracts checklist. | 2 |
| **INTRODUCTION** | | |  |
| Rationale | 3 | Describe the rationale for the review in the context of existing knowledge. | 3-5 |
| Objectives | 4 | Provide an explicit statement of the objective(s) or question(s) the review addresses. | 5 |
| **METHODS** | | |  |
| Eligibility criteria | 5 | Specify the inclusion and exclusion criteria for the review and how studies were grouped for the syntheses. | 6-8 |
| Information sources | 6 | Specify all databases, registers, websites, organisations, reference lists and other sources searched or consulted to identify studies. Specify the date when each source was last searched or consulted. | 5-6 |
| Search strategy | 7 | Present the full search strategies for all databases, registers and websites, including any filters and limits used. | 5-6 and supplementary |
| Selection process | 8 | Specify the methods used to decide whether a study met the inclusion criteria of the review, including how many reviewers screened each record and each report retrieved, whether they worked independently, and if applicable, details of automation tools used in the process. | 7-8 |
| Data collection process | 9 | Specify the methods used to collect data from reports, including how many reviewers collected data from each report, whether they worked independently, any processes for obtaining or confirming data from study investigators, and if applicable, details of automation tools used in the process. | 7-8 |
| Data items | 10a | List and define all outcomes for which data were sought. Specify whether all results that were compatible with each outcome domain in each study were sought (e.g. for all measures, time points, analyses), and if not, the methods used to decide which results to collect. | 7 and supplementary |
|  | 10b | List and define all other variables for which data were sought (e.g. participant and intervention characteristics, funding sources). Describe any assumptions made about any missing or unclear information. | 6-7 and supplementary |
| Study risk of bias assessment | 11 | Specify the methods used to assess risk of bias in the included studies, including details of the tool(s) used, how many reviewers assessed each study and whether they worked independently, and if applicable, details of automation tools used in the process. | 8 and supplementary |
| Effect measures | 12 | Specify for each outcome the effect measure(s) (e.g. risk ratio, mean difference) used in the synthesis or presentation of results. | 8-9 |
| Synthesis methods | 13a | Describe the processes used to decide which studies were eligible for each synthesis (e.g. tabulating the study intervention characteristics and comparing against the planned groups for each synthesis (item #5)). | 8-10 |
|  | 13b | Describe any methods required to prepare the data for presentation or synthesis, such as handling of missing summary statistics, or data conversions. | 9-10 |
|  | 13c | Describe any methods used to tabulate or visually display results of individual studies and syntheses. | 9-10 and 11-12 |
|  | 13d | Describe any methods used to synthesize results and provide a rationale for the choice(s). If meta-analysis was performed, describe the model(s), method(s) to identify the presence and extent of statistical heterogeneity, and software package(s) used. | 8-11 |
|  | 13e | Describe any methods used to explore possible causes of heterogeneity among study results (e.g. subgroup analysis, meta-regression). | NA- possible causes described in discussion p20 |
|  | 13f | Describe any sensitivity analyses conducted to assess robustness of the synthesized results. | 10 |
| Reporting bias assessment | 14 | Describe any methods used to assess risk of bias due to missing results in a synthesis (arising from reporting biases). | MA was performed on complete data |
| Certainty assessment | 15 | Describe any methods used to assess certainty (or confidence) in the body of evidence for an outcome. | 11 |
| **RESULTS** | | |  |
| Study selection | 16a | Describe the results of the search and selection process, from the number of records identified in the search to the number of studies included in the review, ideally using a flow diagram. | 12 |
|  | 16b | Cite studies that might appear to meet the inclusion criteria, but which were excluded, and explain why they were excluded. | 12 |
| Study characteristics | 17 | Cite each included study and present its characteristics. | 12 and supplementary |
| Risk of bias in studies | 18 | Present assessments of risk of bias for each included study. | 13 and supplementary |
| Results of individual studies | 19 | For all outcomes, present, for each study: (a) summary statistics for each group (where appropriate) and (b) an effect estimate and its precision (e.g. confidence/credible interval), ideally using structured tables or plots. | 14-17, and figure2, table 1 |
| Results of syntheses | 20a | For each synthesis, briefly summarise the characteristics and risk of bias among contributing studies. | 13-14 |
|  | 20b | Present results of all statistical syntheses conducted. If meta-analysis was done, present for each the summary estimate and its precision (e.g. confidence/credible interval) and measures of statistical heterogeneity. If comparing groups, describe the direction of the effect. | 15 |
|  | 20c | Present results of all investigations of possible causes of heterogeneity among study results. | Descriptively in discussion p20 |
|  | 20d | Present results of all sensitivity analyses conducted to assess the robustness of the synthesized results. | 14 |
| Reporting biases | 21 | Present assessments of risk of bias due to missing results (arising from reporting biases) for each synthesis assessed. | MA was on complete data |
| Certainty of evidence | 22 | Present assessments of certainty (or confidence) in the body of evidence for each outcome assessed. | 14-15 |
| **DISCUSSION** | | |  |
| Discussion | 23a | Provide a general interpretation of the results in the context of other evidence. | 18-20 |
|  | 23b | Discuss any limitations of the evidence included in the review. | 22-23 |
|  | 23c | Discuss any limitations of the review processes used. | 22-23 |
|  | 23d | Discuss implications of the results for practice, policy, and future research. | 23-24 |
| **OTHER INFORMATION** | | |  |
| Registration and protocol | 24a | Provide registration information for the review, including register name and registration number, or state that the review was not registered. | 5 |
|  | 24b | Indicate where the review protocol can be accessed, or state that a protocol was not prepared. | PROSPERO |
|  | 24c | Describe and explain any amendments to information provided at registration or in the protocol. | No change |
| Support | 25 | Describe sources of financial or non-financial support for the review, and the role of the funders or sponsors in the review. | 24 |
| Competing interests | 26 | Declare any competing interests of review authors. | 24 |
| Availability of data, code and other materials | 27 | Report which of the following are publicly available and where they can be found: template data collection forms; data extracted from included studies; data used for all analyses; analytic code; any other materials used in the review. | All relevant data supporting this study are available in an open repository at https://osf.io/t3cps/. |

*From:* Page MJ, McKenzie JE, Bossuyt PM, Boutron I, Hoffmann TC, Mulrow CD, et al. The PRISMA 2020 statement: an updated guideline for reporting systematic reviews. BMJ 2021;372:n71. doi: 10.1136/bmj.n71

For more information, visit: <http://www.prisma-statement.org/>
